# Unveiling a Dermatological Mystery: A Rare Case of Miliary Cutaneous Tuberculosis in an Adult Patient

**DOI:** 10.7759/cureus.69854

**Published:** 2024-09-21

**Authors:** Cecilia Arias Sepúlveda, Kenia J Hernandez Rivera, Mariana Olaya Cordova, Mariana Gonzalez Plascencia

**Affiliations:** 1 Internal Medicine, Hospital General Presidente Lázaro Cárdenas, Instituto de Seguridad y Servicios Sociales de los Trabajadores del Estado (ISSSTE) Autonomous University of Chihuahua, Chihuahua, MEX

**Keywords:** cutaneous manifestations, cutaneous tuberculosis, disseminated miliary tuberculosis, infectious diseases, mycobacterium tuberculosis

## Abstract

Tuberculosis (TB) continues to pose a significant global health challenge, with Mycobacterium (M.) tuberculosis being the primary pathogen. Cutaneous tuberculosis, particularly in its miliary form, is a rare manifestation, accounting for a small proportion of extrapulmonary cases. We report the case of a 71-year-old male from Chihuahua, Mexico, with a history of smoking, diabetes, and hypertension, who presented with a disseminated polymorphic dermatosis. Histopathological examination revealed granulomas containing Langhans giant cells and acid-fast bacilli, with confirmation of M. tuberculosis through PCR and staining. The patient, in a compromised general condition, was diagnosed with miliary cutaneous tuberculosis and initiated on appropriate therapy. This case emphasizes the rarity of miliary cutaneous TB and highlights the importance of prompt and accurate diagnosis and management, particularly in immunocompromised individuals.

## Introduction

Tuberculosis is a common cause of global mortality. Worldwide, it is caused by mycobacteria, most commonly Mycobacterium (M.) tuberculosis. It is considered by the World Health Organization (WHO) as a global disease, with over 4,000 deaths reported daily, 10.4 million patients infected annually, and 1.5 million deaths annually due to this infection [1]. When it affects the skin, it is called cutaneous tuberculosis. Extrapulmonary tuberculosis is extremely rare. With the increasing incidence of immunocompromised patients, unusual presentations of tuberculosis are observed more frequently. The rarest form of this infection is cutaneous, accounting for 1-1.5% of all extrapulmonary TB manifestations, with a prevalence of only 8.4-13.7% of all TB cases [2,3]. The miliary form presents as a rare entity among all cases of cutaneous tuberculosis [4]. The gold standard for diagnosis remains the identification of M. tuberculosis in a skin biopsy.

In our case, miliary cutaneous tuberculosis was suspected based on clinical presentation and confirmed by biopsy [4,5]. Miliary cutaneous tuberculosis usually occurs secondary to hematogenous dissemination of the infection, often secondary to advanced pulmonary tuberculosis. A case is reported of a patient with miliary cutaneous tuberculosis in an immunosuppressed patient [4].

## Case presentation

**Figure 1:**
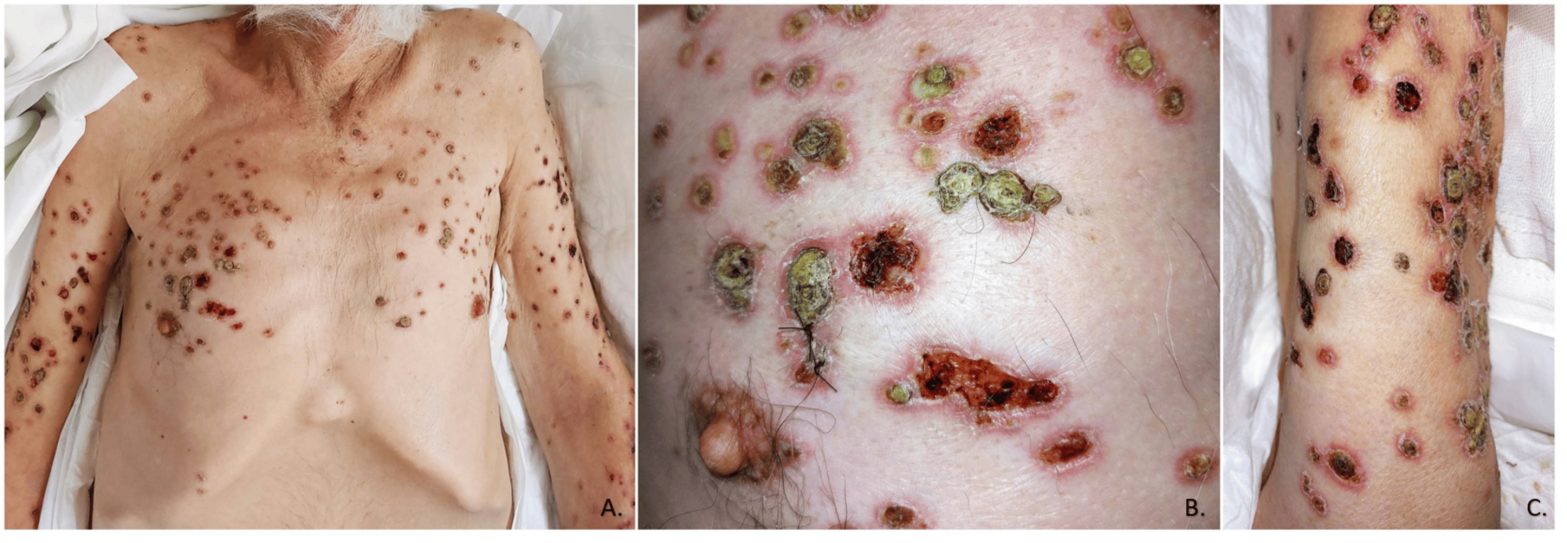
Chest and upper limbs showing polymorphic dermatosis A. Chest and upper limbs showing polymorphic dermatosis, predominantly consisting of round plaques with well-defined borders, ranging from 1 cm to 4 cm in diameter. Some plaques exhibit exulcerated areas and crusts, with erythematous-violaceous borders. B. Photograph with a close-up of the trunk showing the patient's dermatosis and the biopsy suture. C. Close-up photograph of the right upper limb showing the patient's dermatosis.

**Figure 2:**
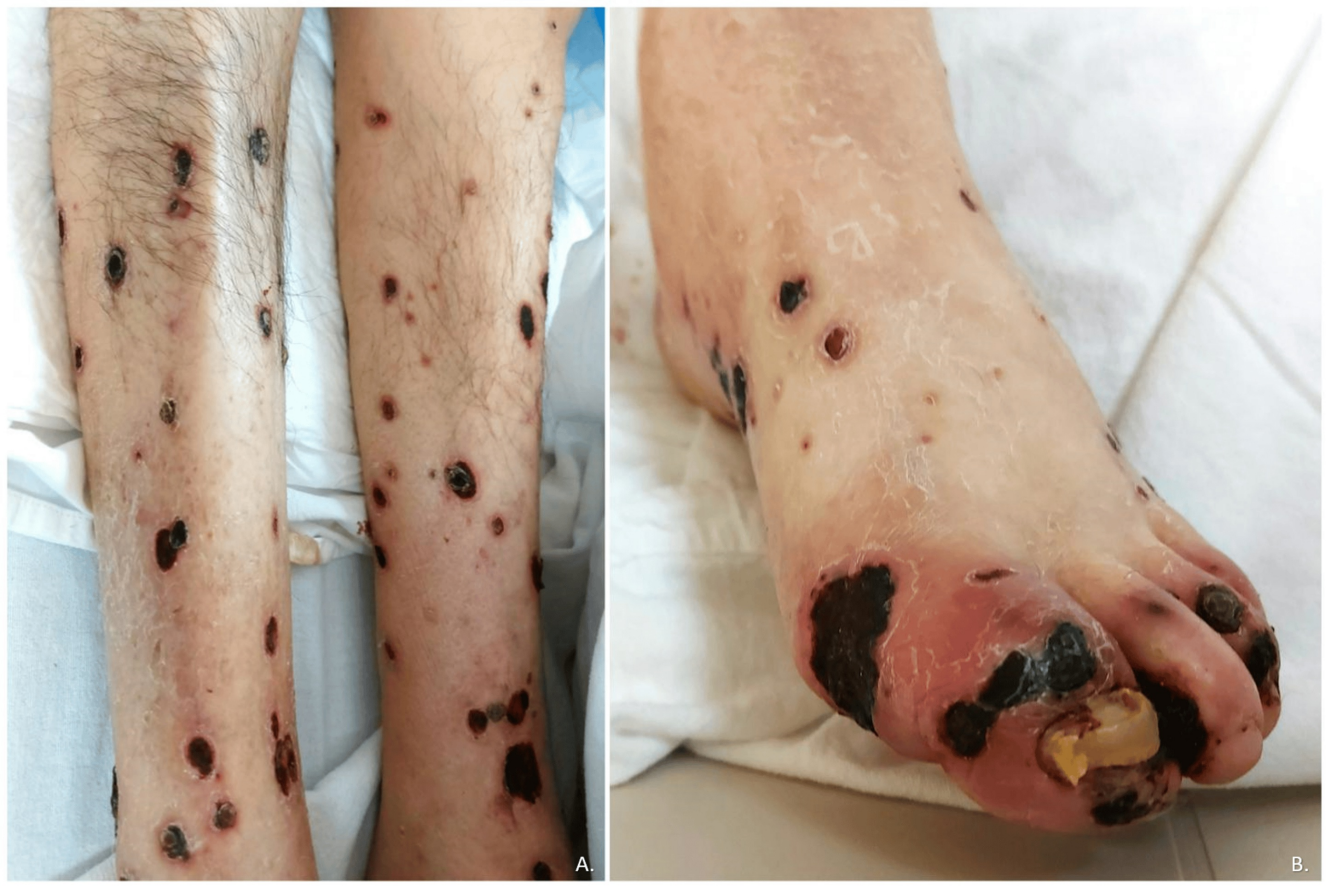
Polymorphic dermatosis predominantly affecting the acral areas A. Polymorphic dermatosis primarily consists of irregular plaques with well-defined borders and eschar, predominantly affecting the acral areas. B. Close-up of the dermatosis previously described on the left foot.

The patient was a 71-year-old male from Junta, Chihuahua, Mexico, employed as an agronomist, with a significant medical history including severe smoking since the age of 15, consuming approximately 30 cigarettes per day, type 2 diabetes mellitus, and systemic arterial hypertension diagnosed 20 years ago, with poor adherence to treatment. One month prior to admission, he was hospitalized at another facility for a gastric ulcer diagnosed via endoscopy. Additionally, he had been experiencing polymorphic dermatosis for two months, characterized by its disseminated and bilateral nature, affecting the head, trunk, and extremities. The dermatosis presented as plaques with defined, irregular borders, and round or oval shapes, ranging from 1 to 4 cm in their largest diameter. These plaques were composed of erythema, with some areas showing ulceration, crusts, and eschar, as shown in Figure 1. On the pelvic limbs and feet, plaques of eschar were noted with greater acral involvement (Figure 2). Ulcers were also present on the hands, characterized by irregular, deep borders exposing muscle tissue and tendons, with erythematous-violaceous edges. Most of the ulcerative lesions were covered with eschar and slough, and multiple larvae were observed.

**Figure 3:**
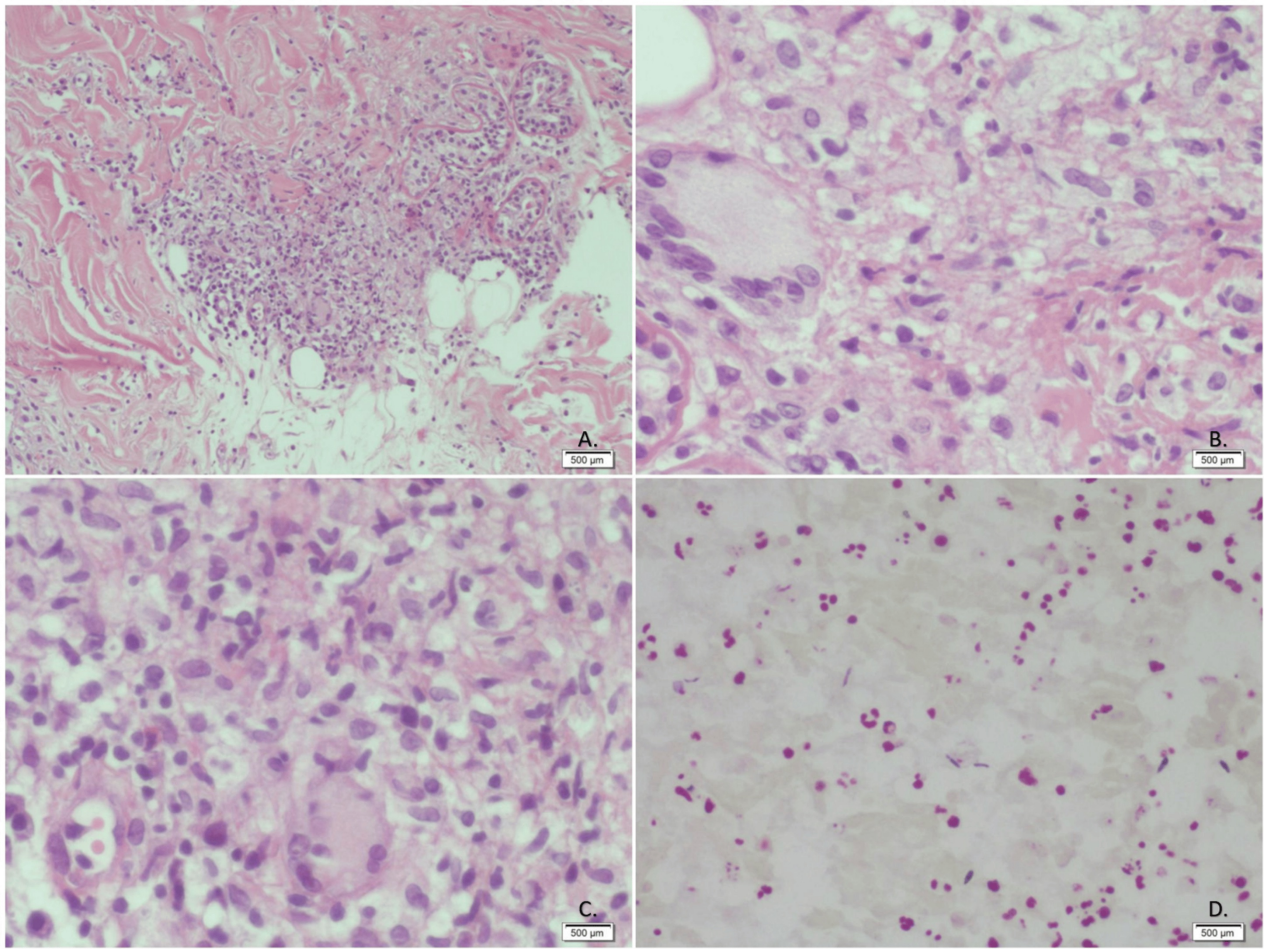
Skin biopsy A. Hematoxylin and Eosin (H&E) stain. A well-formed granuloma is shown with a central area of caseous necrosis, surrounded by a border of epithelioid cells and multinucleated giant cells. This pattern is classic for a Mycobacterium tuberculosis infection. B. Hematoxylin and Eosin (H&E) staining. In this image, multiple multinucleated giant cells are observed, surrounded by an inflammatory infiltrate. These giant cells typically form in response to the presence of foreign bodies or persistent microorganisms such as Mycobacterium tuberculosis. C. Hematoxylin and Eosin (H&E) stain. Dense inflammatory infiltrate is primarily composed of mononuclear cells, such as lymphocytes, macrophages, and some multinucleated giant cells. The giant cells are indicative of a granulomatous response, which is typical of chronic infections like tuberculosis. D. Ziehl-Neelsen staining. Skin. Acid-fast bacilli (AFB) are observed. Images by by Dr. Ernesto Ramos Martínez, Medical Pathologist

The Nikolsky sign was negative, and the oral mucosa remained intact. Microscopic examination revealed that the epidermis exhibited slight hyperkeratosis with orthokeratosis, acanthosis, focal spongiosis, occasional neutrophil exostosis, and vacuolar degeneration of the basal layer. The superficial and reticular dermis showed edema, granulomas, and focal perivascular infiltrates of neutrophils, lymphocytes, plasma cells, and a few eosinophils. The granulomas consisted of epithelial cells, lymphocytes, neutrophils, some plasma cells, and Langhans-type multinucleated giant cells. The hypodermis showed no abnormalities. Ziehl-Neelsen staining and PAS staining revealed Gram-positive bacilli, respectively (Figure 3). Real-time polymerase chain reaction (PCR) testing confirmed the presence of Mycobacterium tuberculosis and excluded atypical mycobacteria.

The pathology report, received 20 days after the biopsy, confirmed the lesion as being caused by M. tuberculosis, and the patient was subsequently transferred to this unit for further management. Upon arrival, the patient was in poor condition, hypotensive, with an oxygen saturation of 88% without supplemental oxygen, cachectic, and exhibiting the previously described disseminated dermatosis. Laboratory results are shown in Table 1.

**Table 1:** Laboratory results

Test	Result	Unit	Reference Range
Hemoglobin	8.1	g/dL	13.8 - 17.2 (men)
Hematocrit	27.8	%	40.7 - 50.3 (men)
Mean corpuscular volume (MCV)	87.1	fL	80 - 100
Mean corpuscular hemglobin (MCH)	25.4	pg	27 - 31
Platelets	370	10³/µL	150 - 450
White blood cells (WBC)	5.04	10³/µL	4.0 - 10.0
Neutrophils	3.78	10³/µL	1.8 - 7.8
Lymphocytes	1.09	10³/µL	1.0 - 4.8
Glucose	253	mg/dL	70 - 99
Uric acid	3.0	mg/dL	3.4 - 7.0 (men)
Urea	68	mg/dL	7 - 20
Blood urea nitrogen (BUN)	31.78	mg/dL	7 - 20
Creatinine	0.7	mg/dL	0.6 - 1.2
Sodium	142	mmol/L	136 - 145
Potassium	4.1	mmol/L	3.5 - 5.0
Chloride	109	mmol/L	98 - 107
Albumin	2.09	g/dL	3.5 - 5.0

**Figure 4:**
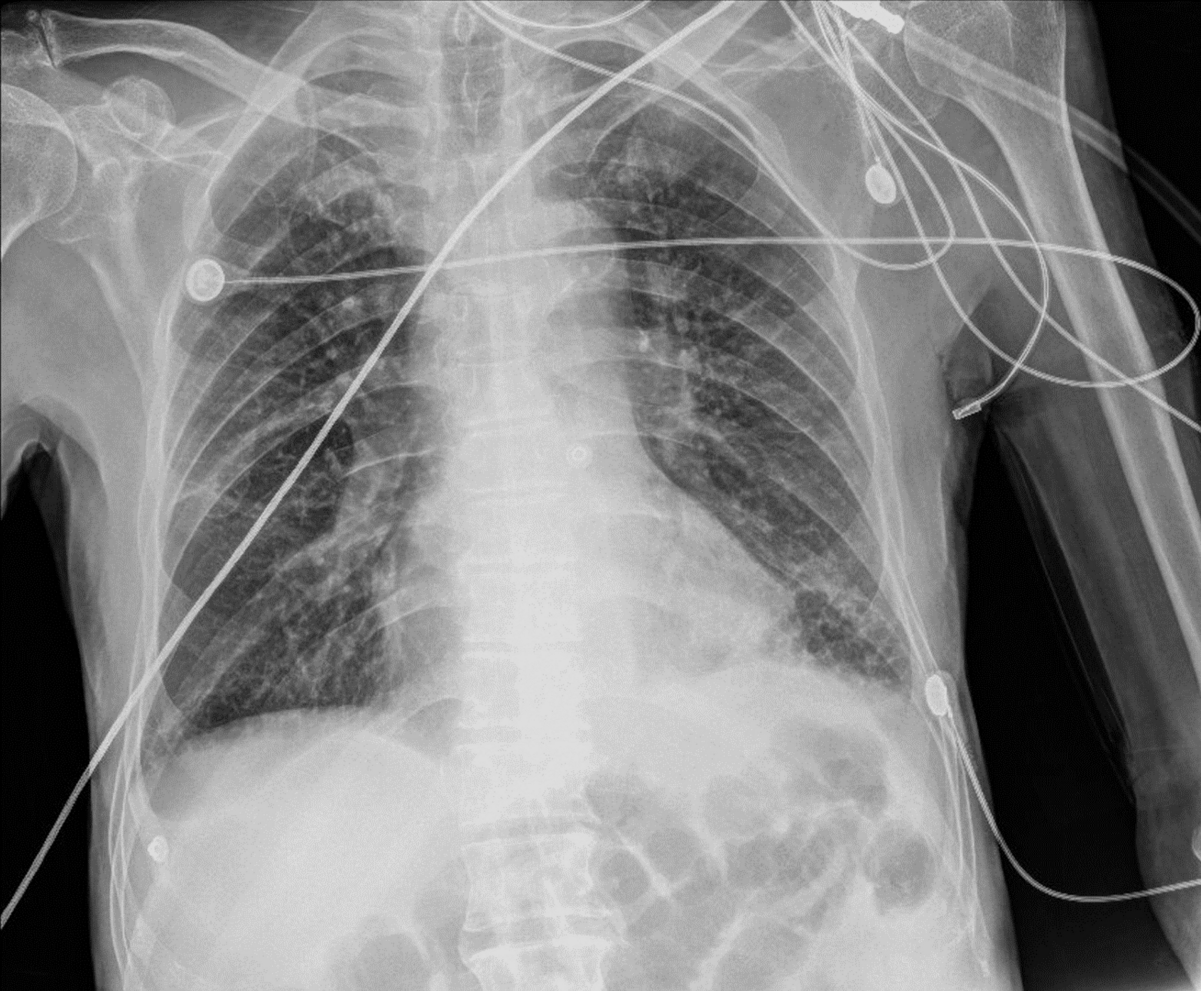
Anteroposterior X-ray showing a diffuse and uniform micronodular pattern in both lungs

The chest X-ray showed a diffuse micronodular pattern in both lungs (Figure 4).

Other conditions of immunosuppression, such as HIV, were ruled out. Differential diagnoses included various maculopapular and purpuric eruptions, including Letterer-Siwe syndrome, acute lichenoid pityriasis, secondary syphilis, and drug reactions.

Given that cutaneous tuberculosis is generally reinfection and that lesions typically develop in individuals with prior pulmonary primary infection, as a primary cutaneous infection is very rare or exceptional, an epidemiological consultation was sought to initiate treatment for cutaneous tuberculosis, which is the same as for systemic tuberculosis. The patient also presented with larvae, indicative of apparent myiasis, diagnosed clinically upon the observation of larvae in both upper extremities. Unfortunately, the patient passed away a few days after admission to this unit.

## Discussion

Disseminated miliary tuberculosis is a rare form of tuberculosis that is seldom observed in immunocompetent patients but occurs more frequently in immunocompromised individuals. In the case of our patient, several factors contributed to the disease's rapid progression despite no documented immunosuppression. His medical history of poorly controlled type 2 diabetes mellitus, long-term smoking, and systemic arterial hypertension likely compromised his immune response, which facilitated the hematogenous dissemination of Mycobacterium tuberculosis. Diabetes, in particular, impairs macrophage and neutrophil function, which plays a critical role in tuberculosis pathogenesis [1,3].

Extrapulmonary tuberculosis represents approximately 20% of tuberculosis cases and includes forms, such as pleural, osteoarticular, cutaneous, genitourinary, pericardial, and meningeal, among others [1]. With the increase in the diagnosis of tuberculosis, the incidence of cutaneous tuberculosis has similarly risen. The epidemiology of cutaneous tuberculosis has not been extensively studied; however, it occurs in countries endemic to M. tuberculosis. Between 1% and 2% of patients with extrapulmonary manifestations suffer from cutaneous tuberculosis [2,3,6,7].

From 1991 to 2002, only 25 cases of miliary tuberculosis were reported worldwide. Although the purified protein derivative (PPD) test is generally positive, it often yields negative results in immunosuppressed patients due to anergy. The first case of miliary tuberculosis was reported in 1990 by Stack et al. [8]. In 1992, Rohatgi et al. reported that cutaneous involvement in miliary tuberculosis is exceedingly rare in patients who are not co-infected with HIV [8]. 

Although various classification systems exist, cutaneous tuberculosis is generally classified according to its morphological pattern, route of acquisition (exogenous, endogenous, contiguous, autoinoculation, or hematogenous dissemination), and the patient’s immune status. It can also be categorized as paucibacillary or multibacillary [1,9]. 

Regarding pathophysiology, endogenous invasion usually results from pulmonary dissemination via hematogenous or lymphatic routes [3,10]. Mycobacterium tuberculosis can be described as a non-capsulated, rod-shaped, non-spore-forming bacillus lacking mobility mechanisms, typically ranging from 1 to 10 µm in length and 0.2 to 0.6 µm in width [2]. Histopathologically, the morphology of lesions can vary. The initial immune response is dominated by neutrophils, followed by NK lymphocytes and macrophages. This neutrophilic infiltrate creates a necrotizing area with an inflammatory infiltrate, and tuberculous bacilli may still be visible in early infections. Macrophages are also among the first lines of defense against tuberculosis. They phagocytize the bacteria, where lysosomal fusion with the phagosome occurs. Bacterial antigens are processed, and macrophages present these antigens to CD4+ T-helper cells through major histocompatibility complex (MHC) class II. Apoptotic vesicles from infected cells also contain antigens and can specifically stimulate CD8+ T cells. Additionally, macrophages secrete IL-12, which induces IFN-γ production by Th1 cells. Subsequently, these macrophages aggregate to form a granuloma around the inoculation area to isolate the bacteria and prevent further spread. Granuloma formation is mediated by tumor necrosis factor-alpha (TNF-α), a pro-inflammatory cytokine secreted by T lymphocytes, monocytes, and macrophages; it maintains granuloma structure by increasing adhesion molecule expression and producing reactive oxygen and nitrogen intermediates [2]. Early lesions may show superficial ulceration, epidermal hyperplasia, and a dense inflammatory infiltrate in the dermis. Over time, granulomatous inflammation replaces neutrophils, with or without caseous necrosis.

The diagnostic process in this case was challenging. While Ziehl-Neelsen and PAS stains confirmed the presence of Gram-positive bacilli, it was PCR testing that definitively identified Mycobacterium tuberculosis and ruled out atypical mycobacteria. Given the polymorphic nature of the dermatosis and the presence of larvae in ulcerated lesions (indicative of myiasis), the clinical picture was complex. This emphasizes the importance of using molecular techniques like serum levels of QuantiFERON-TB Gold (QFT-G) and PCR, especially in cases with atypical presentations where conventional tests may be insufficient [11].

The patient presented with severe polymorphic dermatosis, primarily characterized by ulcerated plaques with eschar, affecting the trunk, extremities, and head. This presentation, particularly the deep ulcers exposing muscle tissue and tendons, aligned with descriptions of cutaneous miliary tuberculosis, which occurs secondary to hematogenous spread from pulmonary or other active foci. Histology of cutaneous lesions in miliary tuberculosis involves nonspecific inflammation with necrotizing vasculitis and tuberculous bacilli, typically surrounded by macrophages and occasionally multinucleated giant cells [3]. In our case, immunosuppression, granulomas, and bacilli in the biopsy led to the confirmation of the microorganism. The manifestation of cutaneous miliary tuberculosis is nonspecific. Other causes of dermatosis such as disseminated infections (bacteria, fungi, herpesviruses, non-tuberculous mycobacteria) must be excluded. Clinically, acute disseminated miliary tuberculosis is characterized by profuse and discrete purpuric papules, often with tiny vesicles that tend to rupture or dry, forming umbilicated scars. Such lesions may heal within one to four weeks, leaving a scar with a halo. Since the diagnosis is confirmed through biopsy, it is strongly recommended to perform biopsies in HIV-positive patients with papulopustular dermatosis and confirm the diagnosis with PCR to detect M. tuberculosis DNA [8].

Despite the timely biopsy confirming the diagnosis, the patient’s clinical condition deteriorated rapidly, indicating the disease's advanced state upon presentation. The patient's severe cachexia, hypotension, and low oxygen saturation were signs of a late-stage disease, where tuberculosis had spread extensively. The presence of myiasis further complicated the prognosis, indicating significant skin barrier disruption and poor overall health. Unfortunately, despite being transferred to the unit for further management, the patient passed away shortly after admission.

Although the treatment for cutaneous tuberculosis typically involves the same first-line drugs used for systemic tuberculosis, including rifampicin, isoniazid, pyrazinamide, and ethambutol, the patient’s critical condition upon arrival likely contributed to the poor outcome. Treatment consists of two phases: an intensive phase aimed at rapidly reducing the M. tuberculosis load and a maintenance phase lasting approximately 9 to 12 months [11]. Isoniazid kills approximately 95% of organisms within the first two days. Rifampicin and pyrazinamide are used during the intensive phase. In the maintenance phase, these drugs are used to kill any remaining sensitive bacilli that were latent during the initial phase [12]. The case underscores the need for early recognition and treatment initiation, especially in patients with pre-existing comorbidities such as diabetes and smoking-related chronic conditions. Moreover, in endemic areas, tuberculosis should remain a top differential diagnosis in chronic dermatoses [12].

## Conclusions

Miliary cutaneous tuberculosis is a rare form of tuberculosis with significant morbidity and potential mortality if not promptly diagnosed and treated. In this case, disseminated miliary tuberculosis complicated by uncontrolled diabetes and myiasis emphasizes the complexity of diagnosing and managing tuberculosis in patients with multiple comorbidities. While cutaneous tuberculosis is rare, this case highlights the importance of maintaining a high index of suspicion for tuberculosis in endemic regions and managing risk factors to prevent severe disease progression. Early biopsy and molecular testing can significantly improve diagnostic accuracy, but as seen in this case, early intervention is critical to improving patient outcomes.
